# Remembering the forgotten non-communicable diseases

**DOI:** 10.1186/s12916-014-0200-8

**Published:** 2014-10-22

**Authors:** Alan D Lopez, Thomas N Williams, Adeera Levin, Marcello Tonelli, Jasvinder A Singh, Peter GJ Burney, Jürgen Rehm, Nora D Volkow, George Koob, Cleusa P Ferri

**Affiliations:** School of Population and Global Health, The University of Melbourne, Building 379, 207 Bouverie St, Carlton, Melbourne, VIC 3053 Australia; Department of Medicine, Imperial College, St Mary’s Hospital, London, W21NY UK; KEMRI/Wellcome Trust Research Programme, PO Box 230, Kilifi, Kenya; University of British Columbia, St Paul’s Hospital, 1081 Burrard Street Rm 6010 A, Vancouver, BC V6Z1Y8 Canada; 7th Floor, TRW Building, 3280 Hospital Drive NW, Calgary, Alberta T2N 4Z6 Canada; Medicine Service and Center for Surgical Medical Acute care Research and Transitions, VA Medical Center, 510, 20th street South, Birmingham, AL FOT 805B USA; Department of Medicine at School of Medicine, and Division of Epidemiology at School of Public Health, University of Alabama, 1720 Second Ave. South, Birmingham, AL 35294-0022 USA; Department of Orthopedic Surgery, Mayo Clinic College of Medicine, 200 1st St SW, Rochester, MN 55905 USA; National Heart and Lung Institute, Imperial College, London, UK; Centre for Addiction and Mental Health, Toronto, Canada; Clinical Psychology and Psychotherapy, Technical Universität Dresden, Dresden, Germany; Addiction Policy, Dalla Lana School of Public Health, University of Toronto (UofT), Toronto, Canada; Department of Psychiatry, Faculty of Medicine, UofT, Toronto, Canada; Institute of Medical Science, UofT, Toronto, Canada; National Institute on Drug Abuse, National Institutes of Health, Rockville, MD USA; National Institute on Alcohol Abuse and Alcoholism, National Institutes of Health, Bethesda, MD 20892-9304 USA; Institute of Education and Health Sciences, Hospital Alemao Oswaldo Cruz, Rua João Julião, 245 – Bloco D CEP 01323-903, São Paulo, SP Brazil; Department of Psychobiology, Escola Paulista de Medicina, Universidade Federal de São Paulo, Rua Botucatu, 862- 1o andar, São Paulo, CEP 04023-062 Brazil

**Keywords:** Global health, Non-communicable diseases, Sickle cell disease, Chronic kidney disease, Asthma, Dementia, Gout, Substance abuse, Alcohol, Liver cirrhosis

## Abstract

The forthcoming post-Millennium Development Goals era will bring about new challenges in global health. Low- and middle-income countries will have to contend with a dual burden of infectious and non-communicable diseases (NCDs). Some of these NCDs, such as neoplasms, COPD, cardiovascular diseases and diabetes, cause much health loss worldwide and are already widely recognised as doing so. However, 55% of the global NCD burden arises from other NCDs, which tend to be ignored in terms of premature mortality and quality of life reduction. Here, experts in some of these ‘forgotten NCDs’ review the clinical impact of these diseases along with the consequences of their ignoring their medical importance, and discuss ways in which they can be given higher global health priority in order to decrease the growing burden of disease and disability.

## Introduction

**Figure 1:**
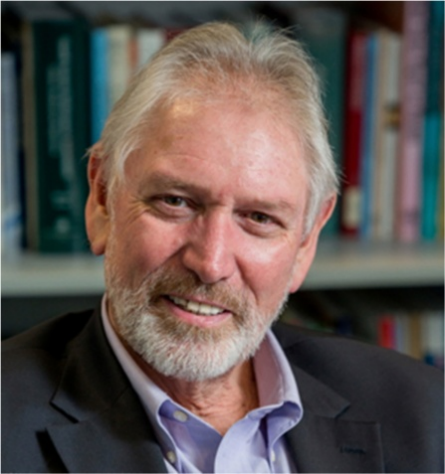
**Alan Lopez is a Melbourne Laureate Professor and the Rowden-White Chair of Global Health and Burden of Disease Measurement at The University of Melbourne.** He is also Director of the Global Burden of Disease Group in the Melbourne School of Population and Global Health.

Alan D. Lopez (Figure 1).

In an era of considerable interest in global health, in part motivated by the Millennium Development Goals, but also inspired by demonstrable success with disease control strategies for child survival, donors, countries and the broader global development community are increasingly asking: what’s next? Certainly, the unfinished agenda of substantially reducing the six million child deaths that still occur each year must remain a focus of global health and development efforts. However, there is now increasing recognition of the imperative not only to keep babies alive until adolescence, but of keeping adolescents alive, and healthy, into old age. Seeing global health priorities as an ‘either/or’ dichotomy is becoming increasingly irrelevant, and uncommon. There is much reference made to the ‘double burden’ or, more correctly, the ‘triple burden’ (including injuries) that low- and middle-income countries are facing. But are we, the global health community, adapting our knowledge base, preventive practices, health care reform and whole-of-government strategies more broadly to cope with what are already the leading causes of health loss, namely non-communicable diseases (NCDs)? Are we doing enough to reduce the significant, but largely ignored, toll that injuries cause throughout the developing world?

Large global descriptive studies of the leading causes of health loss in populations, such as the ongoing Global Burden of Disease Study [1] provide comparable assessments, albeit with substantial and unacceptable uncertainty, of the epidemiological transition that is occurring virtually everywhere in the developing world. They are also able to track the very modest progress that is being made in reducing premature death and disability from injuries, including suicide, homicide and collective violence. Indeed, over 10% of health loss worldwide currently arises from injuries, no different to what it was two decades ago. Meanwhile, the fraction of the global burden of disease and injury due to NCDs, including mental and behavioural disorders, increased from 57% to 65%. In other words, two out of every three years of healthy life lost on the planet are attributable to NCDs. This is not the future; it is the reality of global health today, and it is likely to gather pace.

While demographic factors have contributed substantially to this growth, disease risk has not fallen as rapidly as for leading communicable diseases. There is a very real prospect of rates from major vascular diseases, chronic obstructive pulmonary disease (COPD) and cancers rising in men throughout the developing world during our lifetime as the full effects of their massive uptake of smoking some decades ago begin to be seen [2]. This may well be compounded by the large increases in overweight and obesity that have occurred since the early 1980s, firstly in developed countries, and more recently in many developing populations, leading to substantial increases in disease burden from diabetes [3]. Understandably, much research and many resources worldwide have been invested in understanding the epidemiology of these conditions in order to guide treatment and preventive programs. But, just like the policy focus of the past few decades on child survival, with comparatively little attention to health loss and premature death among adults, one might also ask whether too little attention has been given in global health debates to other NCDs that, for one reason or another, might justifiably deserve more?

**Figure 2:**
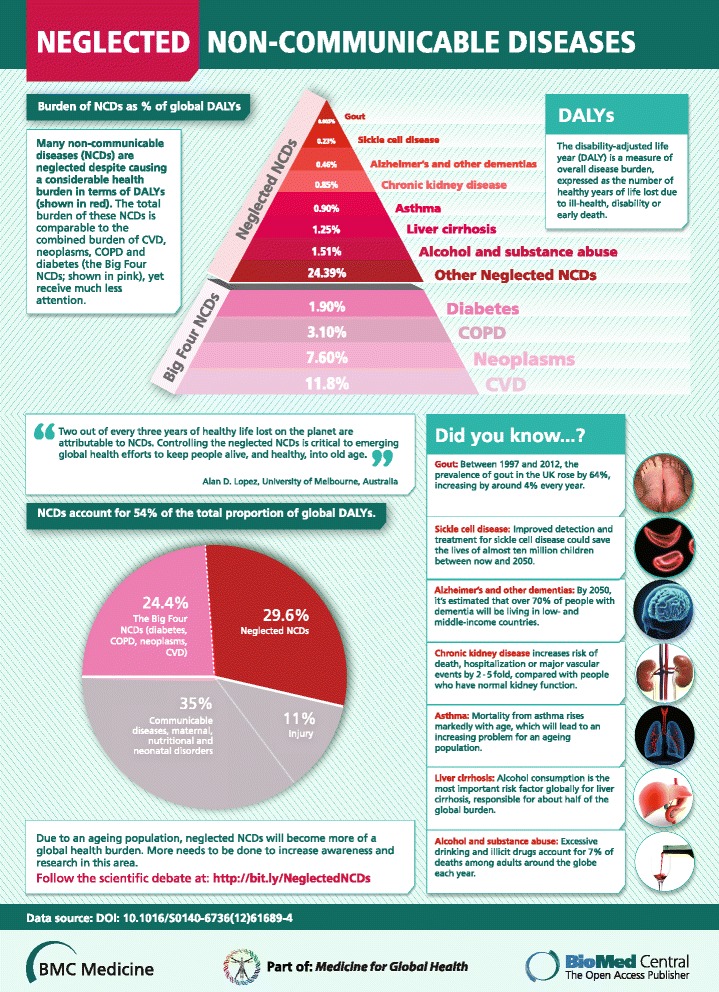
**Comparison of global disease burden (in DALYs) with a focus on neglected non-communicable diseases.** Pyramid: Neoplasms, COPD, cardiovascular diseases and diabetes (the ‘big four’) lead to the highest proportion of disease burden among all NCDs. However, many other NCDs lead to a comparable proportion of disease burden, yet do not receive as much attention as the ‘big four’. We have discussed seven of these neglected NCDs (alcohol and substance abuse, liver cirrhosis, asthma, chronic kidney disease, Alzheimer’s and other dementias, sickle cell disease and gout) and reviewed their disease burden. Pie chart: NCDs account for 54% of total proportion of global DALYs. Although the ‘big four’ comprise just under half of this burden (45% of the burden of NCDs; 24.4% of the total global DALY burden) all other NCDs (i.e. the neglected NCDs) account for 55% of the burden of NCDs; 29.6% of the total global DALY burden. Data for this infographic derived from [13]. The figure has been prepared by BioMed Central.

So what are these ‘forgotten NCDs’ and why do they matter? Just as the creation of the concept (and terminology) of ‘neglected tropical diseases (NTDs)’ has led to much greater recognition, research support and programmatic response, including from institutions such as the Gates Foundation and the World Health Organisation, might a more strategic focus on neglected NCDs engender a similar global response, and is it warranted? The evidence would suggest it is. While neoplasms, COPD, cardiovascular diseases and diabetes cause much health loss worldwide, more of the global NCD burden (55%) arises from other NCDs. These include a diverse set of causes and conditions, but among the more important are musculoskeletal disorders, especially low back and neck pain, depression, substance use disorders, cirrhosis of the liver, chronic kidney disease, asthma, various digestive diseases including peptic ulcer, anxiety disorders, congenital anomalies and haemoglobinopathies. Unlike the ‘big four’ NCDs, many of these conditions cause more health loss through chronic disability rather than premature death; arguably, preventing chronic disability ought to be an important goal of any health system (Figure 2).

The collection of comments in this Medicine for Global Health forum article is a timely reminder that improving population health requires a focus not only on what is important and well-studied, but on what is important and hitherto largely overlooked. Levin and Tonelli point to the urgent need to improve the integration of research across the biomedical and clinical sciences with health systems and population-based studies to enhance policy and patient outcomes for chronic kidney disease. Peter Burney reminds us that the disease burden from asthma is not declining very much at all, and that health services, particularly in poor countries, are ill-equipped to manage the case load, compounded by a poor supply of affordable medicines. Liver cirrhosis, long neglected as a global health priority, is another condition where the etiology is well understood, but as Jürgen Rehm points out, policy responses have been disappointing, particularly in reducing alcohol consumption, a leading risk factor for the disease. More broadly, alcohol and drug use disorders are causing an increasing share of health loss in many populations, quite apart from the social pathologies associated with their use. As Volkow and Koob argue in their article, a more effective response will require a serious rethink of how health care services are provided, with greater emphasis on screening and improved case management.

One of the principal consequences of population aging, namely an increase in dementia, is often at the forefront of policy discussions about the key implications of social, economic and health trends, but there is considerable uncertainty about appropriate policy responses, in part because the condition is not well understood. As Ferri argues, that is changing, with recent evidence emphasizing the importance of primary prevention to reduce this growing disease burden. The lack of visibility for sickle cell disease, as Williams points out, is in part due to poor data, in part due to the fact it is concentrated in the world’s poor, yet the condition accounts for over one-third of the disease burden from haemoglobinopathies. Even gout, though a relatively minor cause of disease burden, is an example of a severely disabling condition that ought, with current knowledge, be better managed and more readily preventable, as detailed in the article by Singh.

Collectively, this reminds us that, unlike the acute, and largely treatable nature of communicable diseases, NCDs are complex, diverse, and manifest their impact on population health in different ways. Mitigating their impact will require a more strategic, comprehensive and balanced approach to NCD research, treatment and prevention, beyond what has been the practice for the past half century or so, giving greater emphasis to those conditions that are major causes of health loss, and which hitherto have been largely ignored as global health priorities. So, what might public health research focus on to accelerate the recognition of neglected NCDs as a global health priority? In my view, four pillars of research and knowledge translation are critical to that endeavour:

i). rapidly reduce ignorance and uncertainty about the true disease burden from these conditions by cost-effectively and strategically improving cause of death and disability data collection systems;
ii). improve knowledge about the most cost-effective strategies to reduce disease burden in different populations, and about the most appropriate and affordable approaches to financing treatment and prevention;
iii). improve knowledge and understanding of established interventions for controlling the impact of the more important forgotten NCDs in health care debates, and promote targeted research on promising intervention options where this is lacking; and
iv). raise the profile of major forgotten NCDs in national and global health fora by developing policy relevant forecasts of likely health, economic and social costs of continuing to ignore them.

We should not continue to ignore or forget these NCDs. The examples reported here suggest the need for an organized, committed and urgent response by the global health community to reducing their disease burden everywhere.

### Competing interests

The author declares he has no competing interests.

### Sickle cell disease: a neglected non-communicable disease of growing global importance

**Figure 3:**
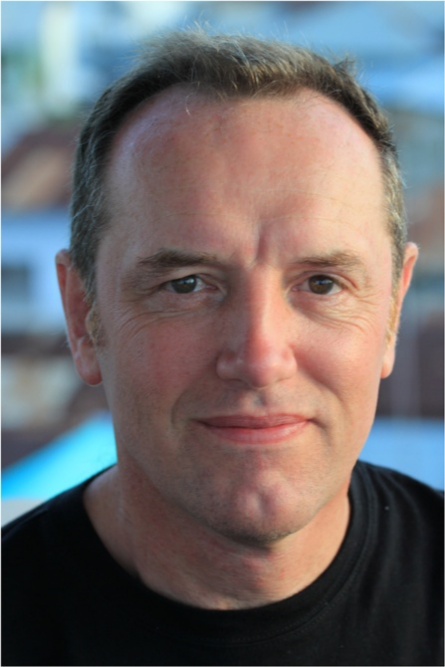
**Tom Williams is Professor of Haemoglobinopathy Research at Imperial College London.** As a clinical academic he has been studying the epidemiology of haemoglobin disorders for more than 20 years, both in terms of the malaria protective effects of carrier forms and the global burden and consequences of homozygosity, particularly in relation to sickle cell disease. Based in Kenya for the last 15 years, he has recognised the growing importance of sickle cell disease as the country has entered its epidemiological transition. He co-chairs the Infectious Diseases Working Group of the Global Sickle Cell Disease Network.

Thomas N Williams (Figure 3).

Few NCDs could be more neglected than sickle cell disease (SCD) [4]: despite the fact that, with early detection and an inexpensive package of basic care, the majority of those born with the condition can expect to lead a good quality of life into late adulthood, most patients with SCD are born in resource-limited settings (RLS) where the vast majority continue to die undiagnosed in early childhood [4,5]. SCD is a haemoglobinopathy, which results from the pathological effects of Haemoglobin S (HbS), an abnormal form of adult haemoglobin (HbA) that results from a mutation (β^s^) in the HBB gene [5]. Most subjects with SCD are β^s^ homozygotes (sickle cell anaemia; SCA), but the condition can also result from the co-inheritance of the β^s^ mutation with a range of other HBB mutations, of which the most common are those that result in HbC and the β-thalassaemias [5]. Despite recent promising developments, including its recognition by both the UN [6] and the WHO [7] as a disease of major and growing importance, for the most part SCD remains virtually invisible on the global health agenda. In common with many neglected NCDs, to a large extent this can be attributed to three interrelated issues: the fact that its greatest burden falls on the world’s poorest communities, the lack of reliable data and inadequate political will.

**Figure 4:**
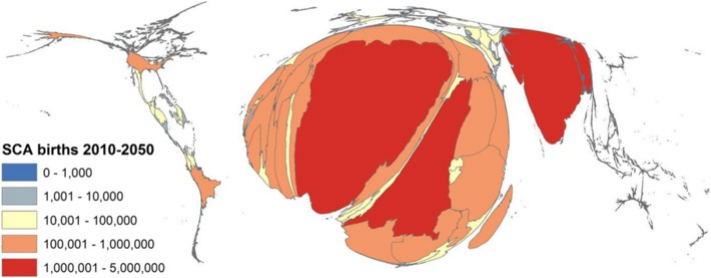
**Cartogram showing the estimated number of newborns with SCA by country.** Cartogram showing the estimated total number of babies that will be born globally, by country, between 2010 and 2050. Figure adapted from Figure 3 within reference [11] drawn and contributed by Dr FB Piel.

Because the carrier state for SCD (sickle cell trait; HbAS) is associated with a strong survival advantage in malaria-endemic areas, the global burden of SCD is also aligned to that of malaria [8]. As a consequence, the vast majority of children with SCD are born in resource-limited settings (RLS) (particularly sub-Saharan Africa) where routine data are least reliable (Figure 4). With few exceptions, diagnostic facilities are poor, early life screening is non-existent and official statistics on health-facility usage and cause of death are sketchy within the RLS in which the prevalence of SCD is highest. The net result is illustrated by the most recent Global Burden of Disease (GBD) Survey [9], a touchstone for policy-makers worldwide, in which causes of death were estimated from vital registration, verbal autopsy (VA), mortality surveillance, censuses, surveys, hospitals, and police and mortuary records. Few reliable data regarding the contribution of SCD to the mortality burden can be derived from any of these sources, exemplified by the fact that before 2012, no specific questions nor any specific diagnostic codes for SCD were included in the standard WHO VA tools [10]. As a result the study grossly under-estimated global SCD-related deaths for 2010 at 28,600 (16,800–40,900) [9], a figure that should almost certainly be 4–6 times higher [11].

Because official statistics are so poor, even basic parameters such as the global number of affected births and SCD-specific morbidity and mortality can only be measured using indirect approaches. For example, we recently used a geostatistical model that combined data on HbAS frequencies, overall birth rates and population densities to estimate birth rates for SCA (which accounts for approximately 70% of SCD) by country, concluding that globally 312,000 (294,000 − 330,000) children were born with SCA in 2010, half being born in just three countries: Nigeria, the Democratic Republic of Congo and India [12] (Figure 4). Similarly, by analyzing population data on the age-specific prevalence of SCA, an indirect measure of the loss through death of subjects with this condition, we recently concluded that current under-5 mortality among children born with SCA in Africa lies between 50% and 90% [4]. As for mortality, the importance of SCD as a cause of global morbidity has been consistently under-estimated through lack of data. Nevertheless, despite this caveat the numbers remain impressive, with estimates from the most recent GBD Survey of 5,641,000 (4,244,000–7,246,000) disability adjusted life years lost [13] and 2,954,000 (1,957,000–4,240,000) years lived with disability [14].

These global figures for morbidity and mortality should be considered in the context of data from the North, where in recent years, many countries have adopted universal screening for SCD and where most now provide comprehensive care for affected individuals. As a result, mortality is now rare among children born with SCD in Europe, the USA and the Caribbean where the majority of affected children can expect to live a relatively normal life into their 40s and 50s [15-17]. Providing such services is within reach in many RLS: successful pilot studies of newborn screening have been conducted in several African countries [18] and, in comparison to diseases of higher priority (such as HIV, TB and malaria), the provision of basic care in specialist clinics is not expensive [19]. If widely implemented, such approaches could save the lives of almost ten million children worldwide between now and 2050 [12].

So how can SCD be brought ‘out of the shadows’ [6] of its current status as a virtually invisible NCD? Perhaps most importantly, we need better data, without which it will remain difficult to persuade ministries of health, policy makers, funders and the pharmaceutical industry to devote appropriate resources to the condition. One essential component is better data on the birth frequencies and survival of children with SCD at the micro-epidemiological level, potentially through investigations using large-scale sample sets collected for other reasons, such as national surveys of micronutrient status, HIV or malaria prevalence. Similarly, the implementation of early-life screening would be made considerably simpler with the development of rapid tests that would circumvent the lack of quality-assured diagnostic laboratories and the logistics of returning results to patients. Better data will lead to better advocacy for SCD at every level: from education in schools and colleges, through to groups of affected patients, the media, celebrities, politicians, funders and health agencies internationally.

### Competing interests

The author declares he has no competing interests.

### Acknowledgements

TNW is funded by a Senior Fellowship from the Wellcome Trust (091758). TNW thanks Dr Frederic B Piel for contributing Figure 4.

### Chronic kidney disease as a global health burden: the need to integrate research and health policy

**Figure 5:**
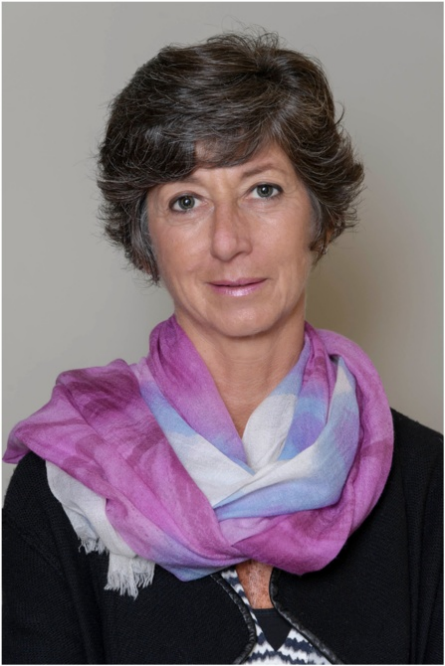
**Adeera Levin is Professor of Medicine and Head of the Division of Nephrology, University of British Columbia, in Vancouver Canada.** She is the Executive Director of the BC Provincial Renal Agency, and President Elect of the International Society of Nephrology.

**Figure 6:**
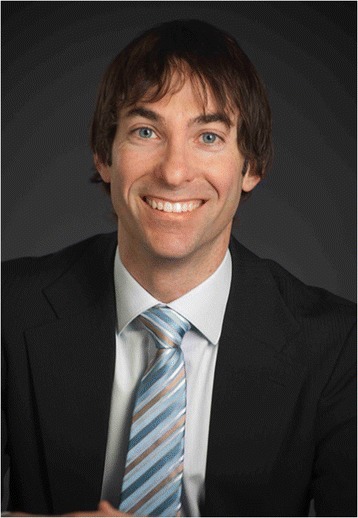
**Marcello Tonelli is Professor of Medicine and Associate Vice President (Health Research) at the University of Calgary in Calgary, Canada.** He is Past President of the Canadian Society of Nephrology and Chair of the Research and Prevention Committee, and council member of the International Society of Nephrology.

Adeera Levin (Figure 5) and Marcello Tonelli (Figure 6).

Chronic kidney disease (CKD) is increasingly acknowledged as a global public health problem, affecting 1 in 10 adults in most jurisdictions [20]. CKD serves as a multiplier of risk in all populations and has a complex interface with other conditions (such as diabetes and cardiovascular disease). The prevalence of risk factors for CKD such as low birth-weight, obesity and hypertension is increasing, and when superimposed on environmental and genetic influences may serve to amplify the rising incidence of CKD over time. In addition, there are specific conditions (such as pregnancy, pre-eclampsia, and acute kidney injury); and specific environments (such as tropical regions, areas of poor sanitation) that may promote or attenuate the progression of CKD [21].

The Lancet publication of the Global Burden of Disease Study 2010 (GBD 2010) serves as important milestone in understanding of population health and disease in this century [9]. The publication highlights the value and power of data to improve our understanding of health, its determinants, and the impact of strategies aimed at addressing specific health issues. Global changes in the incidence and prevalence of key NCDs will continue to impact the incidence of CKD. Further, CKD may influence global metrics of health such as years lived with disability (YLD), given the burden faced by both non-dialysis and dialysis CKD populations [14]. In the GBD 2010 report, deaths due to diabetes increased by 20%, and due to chronic kidney disease by 15% between 1990 and 2010, so that both rose in the ‘league tables’ of causes of death (15 to 9, and 27 to 18 respectively). This change in the relative burden of communicable and non-communicable diseases as drivers of mortality in most countries parallels the profound increase in the ‘lifestyle’ driven risk factors [22]. CKD now ranks 39 globally as a cause of YLD, while diabetes and ischemic heart disease rank 9 and 21 respectively. All of the conditions display regional variability, but note the YLDs due to CKD have increased by 20% since the 1990 report.

As infant mortality and mortality from communicable diseases are reduced in the developing world, the disability from NCDs will increase. Some of that disability will be driven by CKD and all of its consequences. Identifying and implementing proven strategies to address risk factors such as hypertension, obesity, and high salt intake will help to reduce the burden of CKD - as demonstrated in some parts of the world such as Asia [21].

Estimates of the economic burden of CKD vary depending on whether dialysis and transplant therapies are included or excluded. Regardless, it is clear that CKD is a key driver of the high costs associated with NCDs. One report, using provincial data in a Canadian province, described that of a cohort of patients with diabetes, cardiovascular disease and CKD, in various combinations: 18% of hospital costs were attributable to those with CKD either in isolation or combined with either DM or CVD. Thus of this high risk group comprising 7.5% of the total cluster, an annual $ 189M was spent. This study excluded those on dialysis or with transplants, and so is an underestimate of the entire burden [23]. Others have estimated that while the end stage renal disease population (those on dialysis or transplantation) make up less than 1% of the total adult population, they consume up to 5% of national health care budgets [24].

The study and practice of nephrology has changed substantially over the last 50 years. Initially nephrology was a specialty characterized by detailed study of kidney physiology, but has evolved in parallel with availability of dialysis and kidney transplantation – which are no longer experimental treatments, but life-saving therapies that benefit hundreds of thousands of people worldwide. Advances in diagnostics, research and more integrated approaches to care have established CKD as a preventable and treatable chronic disease, with multiple co-morbidities that are directly and indirectly related to CKD. CKD has a dramatic impact on patients and their families - who must live with uncertainty, depression, and the symptoms of kidney disease. Since CKD is a global problem, different health systems, political environments and infrastructure have led to varied strategies and priorities around the world. As an international nephrology community, we recognize that sharing key discoveries, best practices and methodologies is the way forward.

Interventions such as certain drugs, exercise, combined specialty clinics and engagement of primary care and patients, have been studied and shown to improve patient outcomes. Administrative databases are used to understand the impact of CKD on health care systems and generate population-based estimates of disease burden. An increasing number of investigator-initiated randomized trials have begun to address fundamental questions about how best to care for CKD patients - how to prevent or delay kidney failure, when to commence dialysis treatment, how best to treat glomerulonephritis and to prevent rejection of transplanted kidneys. As in other medical researchers, kidney scientists are increasingly interested in new translational approaches to drug development, which may lead to the discovery of novel compounds.

A comprehensive investigative framework that includes four pillars of research (biomedical sciences, clinical research, health systems studies and population research) is the key to improving the outcomes for patients with CKD. The nephrology community has built on this framework to integrate clinical care and health policy with the CKD research agenda. There are multiple examples of teams where clinicians, investigators and administrators work together to improve understanding of the burden in different environments. As an example, there are provincial, national and international specific initiatives whereby researchers, administrators and clinicians collaboratively used data to inform decision making and track outcomes (for example, [25-27]). The CKD PC (Chronic Kidney Disease Prognosis Consortium) has established a rich resource, comprising over one million people with CKD in various stages [28]. The data is collated from interventional trials, large cohort studies and administrative data sets, and as such covers the spectrum of CKD from high risk populations to established CKD populations. Through robust analysis of data, this international group of researchers has established estimates for the prognosis of major events, such cardiovascular disease, hospitalizations, infections, mortality and progression to end stage kidney disease; and recently provided evidence to support a new end point in clinical trials [29]. The latter will facilitate testing of interventions.

Awareness of CKD in the global health arena will depend on continued efforts of the clinical and research communities. The research agenda for nephrology remains multifaceted: the support for basic science discoveries is essential to uncover novel targets and mechanisms to foster drug or therapeutic developments. Scaling up existing administrative, research and clinical databases (some of which have large bio-banking platforms) will optimize the design of clinical trials, and allow clinicians to target the highest risk individuals. New methods for setting research priorities, including the perspectives of administrators, health policy makers, patients and their families, along with practicing clinicians, remains critical. We need new studies that inform evidence-based public policy and assess how best to allocate scarce resources to optimize health outcomes. Finally, there is an increasing emphasis on evaluating the best methods for translation of research findings into practice, so that the science reliably benefits the patients.

CKD will continue to be a major public health problem for the foreseeable future, and the most rapid growth in disease burden will be in developing countries. To achieve a meaningful reduction in death and disability due to CKD, the global kidney research agenda must encompass and integrate basic, clinical, health outcome, and population health perspectives. The international nephrology community is committed to this engagement of patients, healthcare administrators and policymakers in the research agenda. The recognition of CKD as important in the global health and NCD agendas will improve our ability to continue progress and sustain focus on improving patient outcomes - across the continuum of kidney disease and its major risk factors.

### Competing interests

The authors declare they have no competing interests.

### Gout: an Old disease with New windows of opportunities

**Figure 7:**
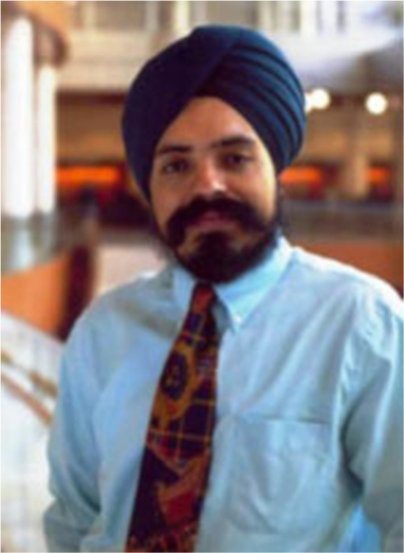
**Jasvinder Singh is an Associate Professor of Medicine at the University of Alabama at Birmingham and a staff physician at the Birmingham Veterans Affairs medical center.** He is an epidemiologist and a clinician with 14 years of experience in treating rheumatic conditions. His research focus is health services and outcomes research in patients with arthritis with a focus on gout, osteoarthritis and arthroplasty. Another area of interest is systematic reviews and meta-analyses, with a focus on Network Meta-analyses. He is the Director of the UAB Cochrane Musculoskeletal Group Satellite Center and serves on several national and International organizations.

Jasvinder A. Singh (Figure 7).

Gout, a common inflammatory arthritis in adults caused by elevated levels of uric acid in the blood that lead to joint inflammation and other manifestations such as kidney stones, is a major public health burden worldwide [30]. Recognized in 2640 B.C., and later described by Hippocrates as ‘the unwalkable disease’, gout is one of the oldest known diseases. It is also a forgotten disease. However, in terms of its impact on patients, high prevalence, well-known pathophysiology and biochemical abnormality, and availability of cheap, affordable treatments, it’s clearly a missed opportunity in medicine.

In the course of several diseases such as diabetes, cancer, rheumatoid arthritis and others, there is a single window of opportunity, where interventions in early disease can prevent future complications. In comparison, gout has several windows of opportunities throughout the disease course and amongst various aspects of the disease. The incidence and prevalence of gout seems to be increasing according to several epidemiological studies. The Rochester Epidemiology project (REP) showed a similar increase in the incidence of gout from 0.045% in 1977–1978 to 0.061% in 1995–1996 [31]. A study in a health maintenance organization showed an almost doubling of prevalence of gout and/or hyperuricemia from 2% in 1990 to 4% in 1999 [32]. A study based on National Health and Nutrition Survey (NHANES) also found that the prevalence of self-reported physician-diagnosis of gout increased from 2.7% in 1988–1994 to 3.9% in 2007–08 [33]. Many of these opportunities may be missed, leading to an increased burden of gout in the face of neglected prospects for diagnosis and treatment.

**Figure 8:**
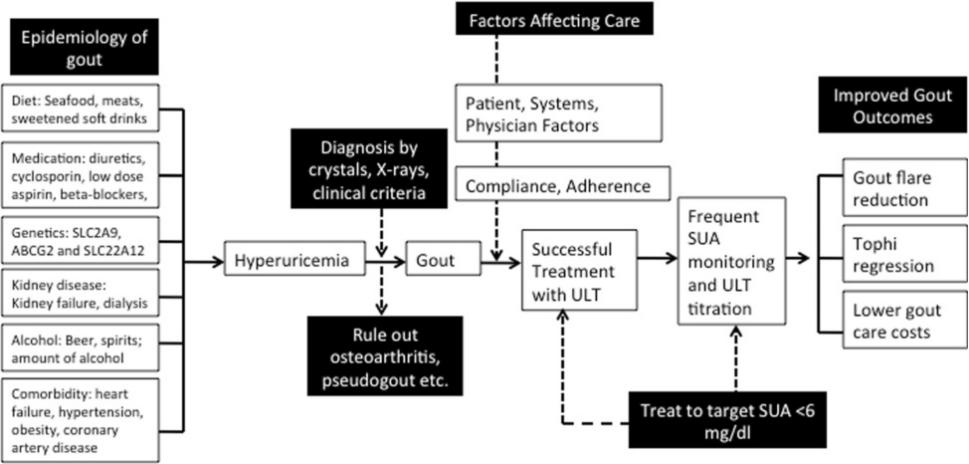
**Epidemiology, diagnosis and optimal management of gout.**

First, physicians and patients need to update their knowledge regarding the dietary and lifestyle risk factors for gout in order to take advantage of these windows of opportunities. New information from well-designed epidemiological studies is available, which update our knowledge of the disease (based on clinical anecdotes) and confirm or refute previous prevalent beliefs about gout. Higher intake of meats, seafood, alcohol (in particular beer) and sugar-sweetened soft drinks (including fruit juices and sodas) increase the risk of gout, while low fat dairy products, Vitamin C supplements and coffee decrease the risk of gout (Figure 8). Importantly, purine-rich vegetables and nuts do not increase the risk of incident gout [34]. A higher intake of purine-rich foods from animal sources (meats etc.) and alcohol increases the risk of gout flares [35,36]. Since environmental factors play a big role in the risk of gout as well as risk of gout flares, they should be one of the main foci of gout management. Our recent work with patients shows that they are interested in discussing these options with their providers as part of their gout management [37,38]. Physicians can counsel patients during their regular follow-up regarding strategies to prevent gout and in those with gout, ways to decrease the risk and suffering from gout flares. Therefore, this is one key area of opportunity for both physicians and patients.

Second is the challenge of correctly diagnosing gout. Gout manifests as intermittent monarticular acute arthritis in the early years/phase of the disease and as chronic polyarthritis with intermittent flares in the later years. Tophi and renal stones may accompany arthritis. The rates of joint fluid aspirate-proven diagnosis of gout are low. Given the common involvement of great toe and other lower extremity joints in two other common conditions, osteoarthritis and pseudogout, a presumptive diagnosis of gout based on a history of big toe pain and a borderline high or higher than normal range serum urate level is problematic (Figure 8). Documentation of additional clinical features of acute synovitis, radiographic signs of overhanging margin and punched-out erosions typical of gout, and close attention to looking for features of other differential diagnoses (osteoarthritis, pseudogout and rheumatoid arthritis), will often help in a correct diagnosis of gout. The 1977 American Rheumatism Association (ARA) preliminary criteria for classification of acute gouty arthritis are also commonly used for a clinical diagnosis of gout, but are inadequate for in-office diagnosis in about 21% of cases [39]. Attempts should always be made to aspirate joint/bursa/tophus and confirm the diagnosis, since documentation of urate crystals in synovial fluid confirms gout as a single test and the treatment for gout is often life-long.

Third is the challenge of optimal control of serum urate. Urate-lowering therapy (ULT), including allopurinol and uricosurics are generic, affordable and the most commonly used drugs; febuxostat, a new ULT, is also available but is more expensive. ULT helps to lower serum urate levels, a central biochemical abnormality in gout. Current guidelines recommend achieving target serum urate of <6 mg/dl [40], which is achieved by at most 33% of patients [41-43]. This is a meaningful disease target since it is associated with improved clinical outcomes such as reduction in gout flares, regression of tophi and lower health care costs (Figure 8) [44-46]. Achievement of this serum urate target frequently requires titration of allopurinol dose, sometimes up to 900 mg/day [47], rather than a monotonic 300 mg/day dose [48]. Physicians aiming to help gout patients avoid flares and improve function to reap the full benefits of treatment need to monitor serum urate after starting ULT and follow a treat-to-target approach. We believe that it is possible to achieve disease remission in many gout patients with this approach. This is a paradigm shift in gout treatment that is likely to improve patient outcomes. A patient-physician collaborative approach is essential for this to succeed.

We have summarized briefly a few opportunities for improving care and outcomes of gout, for which there are several additional opportunities for interventions not mentioned here. We are at the brink of new treatment options for gout, a better understanding of its impact on cardiovascular and renal disease, and better management with existing pharmacological and non-pharmacological treatment approaches. This is an exciting era in which we know ever more about this ancient disease.

### Competing interests

JAS has received research grants from Takeda and Savient and consultant fees from Savient, Takeda, Regeneron and Allergan. JAS is a member of the executive of OMERACT, an organization that develops outcome measures in rheumatology and receives arms-length funding from 36 companies; a member of the American College of Rheumatology's Guidelines Subcommittee of the Quality of Care Committee; and a member of the Veterans Affairs Rheumatology Field Advisory Committee.

### Acknowledgements

JAS is supported by grants from the Agency for Health Quality and Research Center for Education and Research on Therapeutics (AHRQ CERTs) U19 HS021110, National Institute of Arthritis, Musculoskeletal and Skin Diseases (NIAMS) P50 AR060772 and U34 AR062891, National Institute of Aging (NIA) U01 AG018947, National Cancer Institute (NCI) U10 CA149950, and research contract CE-1304-6631 from the Patient Centered Outcomes Research Institute (PCORI). JAS is also supported by the resources and the use of facilities at the VA Medical Center at Birmingham, Alabama, USA. The funding sources (National Institutes of Health and others) had no role in study conception, protocol development, data analyses, manuscript preparation or decision to submit.

### Asthma: a challenge for health care providers

**Figure 9:**
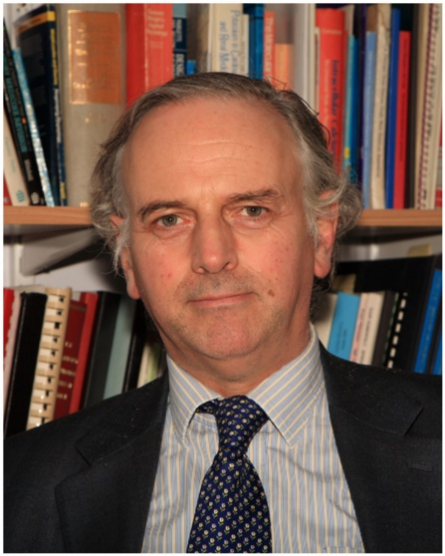
**Peter Burney is Professor of Respiratory Epidemiology and Public Health at the National Heart and Lung Institute, Imperial College London.** Until 2006 he was Chair of Public Health and Primary Care at King’s College London. In the late 1980s he started the European Community Respiratory Health Survey to study asthma and allergies in adults, mostly in Western Europe. Currently he co-ordinates the Burden of Obstructive Lung Disease Study, a study of chronic obstructive lung disease mostly in low and middle income countries.

Peter G. J. Burney (Figure 9).

Asthma is generally defined as a reversible obstruction of the airway and is one of the most common chronic conditions. Since the 1980s the term has come increasingly to signify any wheezy illness that responds to bronchodilators. Compared with other chronic lung diseases it starts much younger and because the mortality is relatively low and the disease tends to persist, it maintains a high prevalence in the population.

Asthma is generally divided into allergic and non-allergic. The relation of asthma to allergic sensitisation is complicated. Both sensitisation and atopic diseases such as asthma run in families, but they are not inherited in the same way [49,50]. In childhood, asthma associated with allergies is more persistent and tends to be more severe. In adulthood ‘non-allergic’ asthma tends to be more severe. In childhood allergies are less common in low income countries, as are the atopic conditions associated with allergies, but non-allergic wheeze is equally common in countries at all economic levels [51]. The prevalence of allergic sensitisation has been increasing over the long term [52], though more recent changes in the prevalence of atopic conditions such as asthma have been more variable among children [53].

The International Study of Asthma and Allergies in Childhood (ISAAC) Study was the largest global survey of the prevalence of asthma, rhinitis and eczema involving almost 2,000,000 children in 105 countries. This study has shown very wide variations in the prevalence of wheezy illness, with very high rates in the English speaking countries and Latin America and wide variations even across single continents such as Europe, where rates fall from high levels in the northwest to low levels in the southeast [54]. The prevalence of more severe disease has a different distribution, however, with a far higher proportion of cases being recorded as severe in sub-Saharan Africa in particular [55].

Information on adults is more sparse and comes from the European Community Respiratory Health Survey (ECRHS) and the World Health Survey [56]. Although mean prevalence is least common in middle-income countries, the maximum prevalence recorded in the poorest countries is below the maximum prevalence in middle or high income countries. Sampling decisions need to inform the interpretation of all these studies. For instance, in low income countries there has been a consistent finding that asthma is less common in rural areas [57-59] and over sampling of urban populations may therefore inflate overall estimates in low income countries.

Asthma is not a common cause of death and age-standardised death rates fell by 42% between 1990 and 2010 from 9.0 to 5.2 per 100,000. However, the global number of deaths fell only 9% from 380,000 to 346,000 between 1990 and 2010 [22]. The slower rate of decline in total deaths represents the aging of the population. Mortality from asthma increases markedly with age and with age some patients with asthma experience a marked decline in lung function [60]. This will lead to an increasing problem for an ageing population whatever changes in prevalence occur. The burden associated with asthma is therefore likely to increase both because of continuing urbanisation in the poorer countries and because of the ageing of the population everywhere.

Currently there is wide variation in the relative impact of asthma on mortality. Although asthma ranks as only the 42^nd^ most common cause of death globally, it is much more highly ranked in Oceania (13^th^), South East Asia (25th), South Asia (26^th^) and North Africa and the Middle East (30^th^) than in Southern and Andean (65^th^ and 62^nd^) Latin America and Western Europe (60^th^).

Because asthma often has an early onset and is persistent throughout life, it is a relatively important cause of disability adjusted life years lost (DALYs), ranking 28^th^ globally among the causes of DALYs but 8^th^ in Oceania, 15^th^ in Australasia and Tropical Latin America, 18^th^ is South East Asia and 19^th^ in the Caribbean [13].

The chronic nature of asthma requires continuous care and reliable access to affordable medications. These conditions have been set out by the Global Initiative for Asthma (GINA) [61] together with the need to prevent exacerbations with the use, in the first instance, of inhaled corticosteroids. However the costs and availability of inhaled steroids are very variable and there is a tendency for these to cost more in low income countries [62]. This leads to poor management and reliance on emergency rooms to provide care, a wasteful and less effective method of managing the condition. In a survey of treatment failures seen in emergency rooms in 11 countries, patients with inadequate insurance and those without a consistent source of continuing medical care were less likely to be on the recommended dose of inhaled steroids. In addition, those without adequate steroid use were more likely to have lost work because of asthma in the recent past [63], demonstrating the high cost to patients and their families of inappropriate care.

Asthma is a common condition that causes considerable morbidity and with increasing age leads to a disease that is more difficult to manage, along with increasing mortality. Although it is mostly not difficult to manage, along with other chronic conditions it requires continuous care, which traditional health services are not designed to provide. Currently the problem of inadequate health services is compounded by a poor supply of over-priced medications. These problems are shared by other chronic conditions and have common solutions including the provision of continuous long-term care and reliable access to affordable, high-quality medication.

### Competing interests

PGJB has acted as a Consultant to Novartis through Imperial Consultants.

### Liver cirrhosis – time for addressing a neglected non-communicable chronic disease

**Figure 10:**
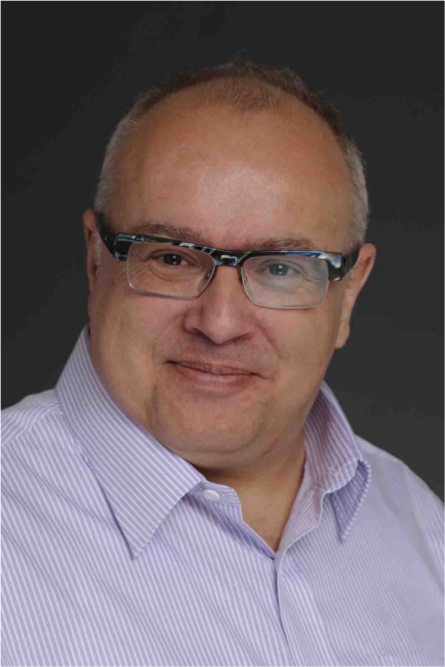
**Jürgen Rehm, Ph.D. has been appointed the Inaugural Chair for Addiction Policy at the Dalla Lana School of Public Health of the University of Toronto.** In addition he holds positions at the Centre for Addiction and Mental Health (Toronto, Canada) as Director of the Social and Epidemiological Research Department and Head of the PAHO WHO Collaborating Centre, and at the Institute for Clinical Psychology and Psychotherapy of the Technical University Dresden (Germany). Dr. Rehm has published more than 600 peer-reviewed publications in addiction research, comprising studies in epidemiology, economics and clinical research, the latter especially in the area of treatment evaluation. He is listed among the ISI/Thompson Reuters most highly cited in the fields of social research and epidemiology and has been awarded the Jellinek Award, the most prestigious award in alcohol research. He has served as public health consultant to many countries, and is currently member of the WHO Expert Advisory Panel on Drug Dependence and Alcohol Problems.

Jürgen Rehm (Figure 10).

Liver cirrhosis is an abnormal condition with irreversible scarring as a result of continuous and long-term liver damage, which is primarily caused by excessive alcohol consumption, hepatitis, and non-alcoholic steatohepatitis. It is among the top 15 causes of death globally and, in 2012, was estimated to have caused more than 1,000,000 deaths and more than 36,000,000 years of lives lost to either premature death or disability.

Global death rates due to liver cirrhosis seem to have been quite stable over the years: the World Health Organization (WHO) estimated and predicted 14.5 deaths per 100,000 for the years 2000 and 2030, respectively, with almost no variation for years in between [64,65]. However, when standardized rates are considered, liver cirrhosis deaths are predicted to decrease.

There are several factors that may become important to explain trends for liver cirrhosis. A downward trend in high income countries may be predicted to be linked to improved clinical practices leading to lower case fatality rates [66,67], although there is not much evidence on this, and, to give just one example, historically, case fatality rates have not shown any improvement over the time period between 1968 and 1999 in England [68]. An upward trend may be linked to increases in alcohol consumption in low- and mid-income countries as they increase their economic wealth [69]. In terms of burden of disease, most DALYs were derived from years of life lost to mortality, that is, due to the high case fatality. However, as indicated above, this may constitute an underestimate for high-income countries due to the lower case fatality and thus higher duration of living with disability.

While the overall prevalence of liver cirrhosis mortality and burden of disease seems stable, there are huge variations by gender, age and regions [70], and they seem to be caused by preventable risk factors. Men have considerably higher rates of liver cirrhosis morality and burden of disease, globally more than twofold the rates of women, and highest in the men between 50 and 69 years of age. The only exception for the higher prevalence of liver cirrhosis mortality and burden is the Eastern Mediterranean region, where women have slightly higher rates; this region also has by far the lowest alcohol consumption [71], which is the main risk factor for liver cirrhosis.

**Figure 11:**
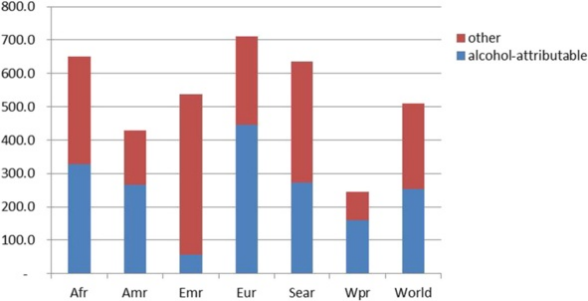
**Disability adjusted life years due to liver cirrhosis per 100,000 population in WHO regions in 2012.** Afr: African region. Amr: Americas. Emr: Eastern Mediterranean region. Eur: Europe. Sear: South East Asian Region (including India). Wpr: Western Pacific Region.

Figure 11 gives an overview of the burden of disease of liver cirrhosis for the year 2012 in DALYs by WHO region, and the role of alcohol in causing this disease (data based on [71,72]). As indicated above, alcohol consumption is globally the most important risk factor for liver cirrhosis, responsible for about half of the global burden (50%; men: 53%; women: 44%; [71]); other main global risk factors are hepatitis B and C, and obesity [13,73]. Europe, especially the Eastern European region, has the highest rate of liver cirrhosis, with alcohol consumption a large factor (63%). For low- and mid-income countries, hepatitis-induced liver cirrhosis is relatively more important, with the relative impact differential between high income and other countries being largest for hepatitis B [13,74].

The role of alcohol consumption in causing and worsening the course of liver cirrhosis has been evident on the individual level [3] and on the aggregate level in comparisons between countries [75], or in analyses over time [76]. It is important to understand that alcohol consumption increases the risk of mortality for all kinds of liver cirrhosis, independent of the original aetiology (3), and thus abstinence is the major goal in most guidelines for treatment of liver cirrhosis.

Intervention studies show that a reduction of alcohol consumption via policy interventions resulted in a marked reduction of liver cirrhosis (for example in Russia [77,78]). Although liver cirrhosis is a chronic disease, interventions have immediate effects, as not only shown by the Russian experiences cited above, but also for instance by the impact of the German seizures of alcohol on French mortality rates during World War II, or the impact of prohibition in the US [75,79,80]). It may take up to 20 years, however, before all of the effects of interventions can be seen [81].

A sizable portion of liver cirrhosis mortality could be reduced in the first year after implementing effective interventions to reduce alcohol, such as higher taxation, decreased availability, advertisement and marketing bans or brief interventions and treatment [82,83], especially among heavy drinkers [72,84]. The effect is more pronounced for heavy drinkers because the risk curve for mortality is exponential; that is, relatively more mortality can be avoided for the same amount of reduction in average drinking for heavy drinkers compared to light or moderate drinkers [72,84]. Such interventions would not only reduce liver cirrhosis rates but also other causes of mortality such as other non-communicable diseases (cancers, hypertensive heart disease, stroke, pancreatitis) or injuries [71,82,84,85]. Given this situation, and given the fact that there are proven effective interventions to reduce alcohol consumption, we see no reason why global liver cirrhosis rates should continue to be as high as they are now.

### Competing interests

The author declares he has no competing interests.

### Acknowledgements

I would like to thank Drs. Gretchen Stevens, Michael Livingstone and Robin Room, who provided important information for this article.

### Substance use disorders: implications for global health

**Figure 12:**
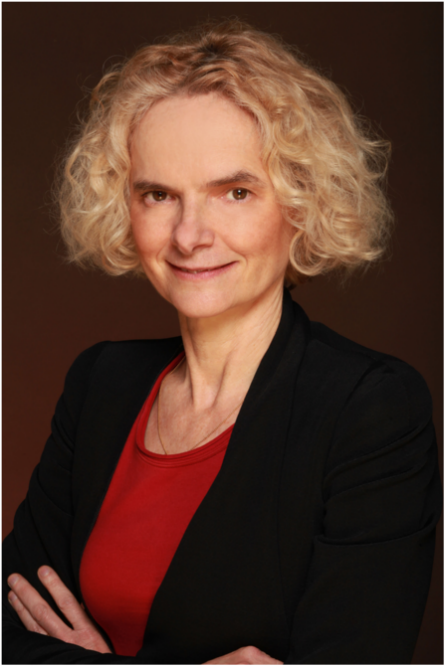
**Nora D Volkow is Director of the National Institute on Drug Abuse; a position she had held since 2003.** Her research transformed the drug addiction field by providing the first evidence for specific molecular (loss of striatal D2 receptors) and functional (impaired frontal control circuitry) changes in brains of addicted individuals that link to compulsivity and loss of control. She has also made ground-breaking discoveries in the neurobiology of ADHD and obesity.

**Figure 13:**
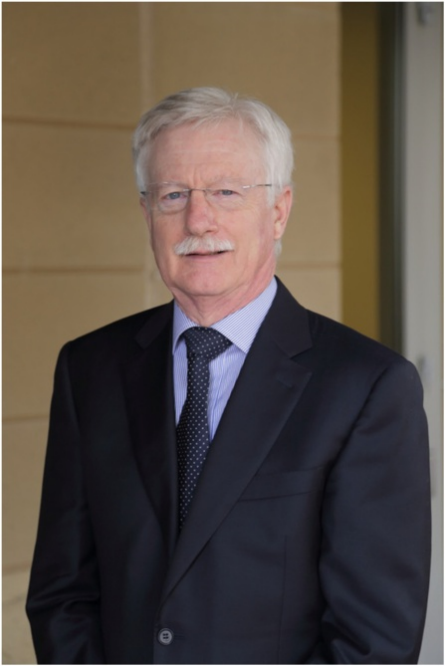
**George F. Koob was recently appointed Director of the National Institute on Alcohol Abuse and Alcoholism after 30 years at The Scripps Research Institute in La Jolla California.** His research has focused on the dysregulation of the brain arousal and stress systems that drive compulsive drug and alcohol seeking. He has made significant contributions to our understanding of the neurocircuitry of negative emotional states and their role in pathophysiology.

Nora D Volkow (Figure 12) and George Koob (Figure 13).

Substance use disorders (SUD) associated with legal substances are two of the three leading contributing factors for global burden of disease and injury (DALYs for tobacco: 6.3%; for alcohol: 5.5%) [9] and those associated with illicit substances are within the top twenty factors (DALYs 0.8%) [86]. The past 20 years has seen an increase in the contribution of SUD to the global burden of disease, mostly from alcohol (32%) and illicit drugs (57%) [9]. Moreover, the global burden of disease attributable to SUD is likely to be underestimated particularly for illicit substances due to incomplete epidemiological data on estimates of impactful and preventable outcomes (i.e. injuries, violence and mental health problems) [86]. Thus, SUV prevention and treatment would have a major impact in improving public health globally.

The recent endorsement by the United Nations Office on Drugs and Crime (UNODC) of addiction as a brain disease and the recommendation that it should be treated as a medical and public health issue rather than a criminal justice and or moral issue highlights the role that the healthcare system can play in the prevention and treatment of SUD [87]. The conceptualization of addiction as a brain disease reflects in part findings from brain imaging studies and preclinical research that have identified the brain circuits that are disrupted by drugs (legal and illegal) and how their disruption impairs the addicted individual’s ability to control his/her behaviour [88]. Moreover, excessive drug and alcohol use in adolescence impairs executive function and increases the vulnerability to SUDs in adulthood. Clinical studies have also shown that SUD can be prevented and treated, and like other chronic diseases requires continuity of care [89]. Because all countries have health care infrastructure, it is recommended that these healthcare systems integrate treatment of SUD within the system norms. Drug associated health consequences are still some of the main preventable causes of disability and the healthcare system can play a crucial role in their prevention and treatment. This recommendation provides a platform that is relevant and available to countries with different levels of economic development.

Health care systems can participate at all levels in the severity range of SUD, starting from its prevention to serving as a referral for specialized care for the most severe cases. Health care systems can also maximize the opportunity to integrate the care for the health problems associated with SUD. Of particular importance is the management of mental illnesses, since they are frequently co-morbid with SUD, and inappropriate management of either condition exacerbates the other. Similarly, integrated care is fundamental for the treatment of infectious diseases such as HIV and HCV for which substance abusers are at higher risk and for which compliance with medical treatment of the infectious disease requires parallel treatment of the SUD. In addition, SUD is the main underlying cause of vehicle accidents. Therefore, integrated care will also facilitate addressing this factor, for if untreated the alcoholic or drug abuser will continue to contribute to repeated incidents. Moreover, in these times of increasing health care costs and burden, treating SUD’s would translate into significant savings in the need to treat the secondary health costs of SUD.

The challenges in implementing healthcare involvement in SUD management are complex and will vary among countries on the basis of their economic resources, cultural norms, drug availability and policies towards criminalization and legalization of drugs. This is further compounded by rapid changes in the use of drugs across the world, such as movements towards legalization of marijuana, recent access to electronic drug delivery devices, rapid dissemination of new synthetic drugs and the increased abuse of prescription medications. The opportunities that the healthcare system offers towards the control of SUD highlight the urgent need for educating health care providers in the screening and management of SUD and the need to allocate the resources necessary for its implementation.

### Competing interests

The authors declare they have no competing interests.

### Placing dementia in the NCDs prevention strategies

**Figure 14:**
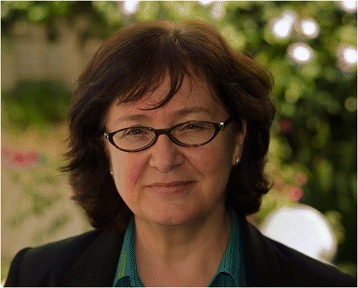
**Cleusa P. Ferri is an Affiliated Professor at the Universidade Federal de Sao Paulo in the post-graduation program of the Psychobiology Department, supported by Associação Fundo de Incentivo a Pesquisa (AFIP), and a Senior Epidemiologist at the Institute of Education and Health Sciences at the Hospital Alemao Oswaldo Cruz, Brazil.** She worked as an international specialist on dementia for the Global Burden of Disease 2010 Project. In the same capacity, she was also involved with the MHGap project with the WHO. For 10 years Dr Ferri worked at the Institute of Psychiatry, King's College London. During this period, apart from her teaching and other research activities, she worked with the 10/66 Dementia Research Group, studying the epidemiology of dementia in low- and middle-income countries. She returned to Brazil, her home country, in 2013 and is now focusing her work on the epidemiology of ageing and dementia in Brazil and Latin America.

Cleusa P. Ferri (Figure 14).

Dementia is a syndrome that usually involves with loss of memory, reasoning, and other cognitive functions progressively impairing an individual’s everyday functioning. The main risk factor for dementia is age, with prevalence roughly doubling every 5 years over the age of 65. Older people are likely to have multiple health conditions. Dementia however, has a disproportionate impact on independent living, being a major cause of disability and dependence [90,91] among older people. With the rapid ageing of the population worldwide, the number of people with dementia is predicted to rise. It is estimated that in 2010 there were 35.6 million people with dementia, and predictions based on population ageing show that this figure is likely to double every 20 years, reaching 115.4 million by 2050 [92]. Most people with dementia already live in low- and middle-income countries (LMIC) and these same estimates predict that by 2050 more than 70% of people with dementia will be living in these countries [92].

It has been estimated that the total worldwide cost of dementia was US$604 billion in 2010 [93]. While most of these costs are concentrated in high income countries, where the costs are divided roughly equally between formal (hospital and social) and informal (family) care settings, in low- and middle-income countries, informal care costs account for the vast majority of total costs [93], with the burden concentrated on families and informal carers. Formal costs are likely to increase in these countries, not only due to the increasing numbers of people with dementia in the future, but also because of a shift in the balance between informal and formal care as the health care sectors develops in LMIC.

Future estimates are mostly based on population ageing and include the assumption that age-specific prevalence will be stable over time. However, some recent studies have suggested that over the past 20 to 30 years there has been a decline in the predicted burden of dementia in high-income countries [94-97]. In the UK, for example, a recent study [95] indicates a reduction in dementia prevalence of around 20% over a 20-year period (from 1989–1994 to 2008–11). These reductions suggest that predicted dementia cases were perhaps avoided or delayed by changes in the risk factors for dementia at earlier ages, suggesting that these risks are modifiable and dementia, to some extent, can be prevented or, at least, the risk reduced at particular ages.

One possible explanation for the reduction in dementia is the change in cardiovascular diseases and risk factors. There is growing evidence supporting a strong and likely causal association between cardiovascular disease (CVD) and its risk factors, and dementia [98]. Therefore, the changes seen regarding the reduction in dementia prevalence are likely partially due to improvements in service provision and disease management of CVD, and also to changes in behaviour, with around half of the reduction in morbidity and mortality thought to be accounted for by primary prevention. However, current models to estimate the impact of preventive strategies on future vascular diseases do not consider the impact on dementia. Some non-communicable diseases (NCDs), such as cardiovascular diseases, are risk factors for dementia and many risk and protective factors for dementia are the same as those for other NCDs [99]. However, most countries’ policies and prevention strategies for NCDs do not include the prevention, or reduction of risk, of dementia, despite some recent initiatives [99].

In low-, middle- and high-income countries, dementia can be seen as part of normal ageing. Although dementia is indeed common in the oldest age groups, it is not an inevitable consequence of long lifespans. Increasing awareness of dementia in society as whole, from patients to policy/decision makers, can contribute not only to decreasing stigma and increasing community solidarity, but also to improving the capacity of existing services with evidence based approaches that can meet the needs of older people and those with dementia. It is important to note that awareness campaigns need to be carried out with great care in order to avoid raising expectations that cannot be met, and avoid leading to unintended consequences, such as increased stigma and fear, through use of dramatic imagery and language.

Dementia costs to individuals, families and society as a whole will grow as the number of people with dementia increases, and this will have an even greater impact on LMIC, which have fewer resources and where population ageing is happening faster than in rich countries. Dementia should be on the public health agenda of each country, with careful consideration given to each country’s demographic and sociocultural context, including their own stage of the unfolding demographic and health transitions. The impact of change to risk factor profiles in countries is difficult to predict. However, models looking at the relative impact of primary prevention in comparison to approaches focused more on secondary prevention suggest that up-stream primary prevention is likely to be the cheapest and most efficient way to decrease the burden of dementia for future generations [100-102], reducing the need for costly screening and treatment regimens for established disease. It is important to strengthen the evidence on the effectiveness of dementia prevention programmes, including their timescales, and also to ensure that dementia takes its place in NCD policies and prevention strategies.

### Conflict of interest

Cleusa Ferri has been named as a local principal investigator in an upcoming trial sponsored by Merck.
